# Exploring Natural Clusters of Chronic Migraine Phenotypes: A Cross-Sectional Clinical Study

**DOI:** 10.1038/s41598-020-59738-1

**Published:** 2020-02-18

**Authors:** Yohannes W. Woldeamanuel, Bharati M. Sanjanwala, Addie M. Peretz, Robert P. Cowan

**Affiliations:** Stanford Headache and Facial Pain Division, Department of Neurology and Neurological Sciences, Stanford University School of Medicine, Stanford, CA USA

**Keywords:** Migraine, Migraine

## Abstract

Heterogeneity in chronic migraine (CM) presents significant challenge for diagnosis, management, and clinical trials. To explore naturally occurring clusters of CM, we utilized data reduction methods on migraine-related clinical dataset. Hierarchical agglomerative clustering and principal component analyses (PCA) were conducted to identify natural clusters in 100 CM patients using 14 migraine-related clinical variables. Three major clusters were identified. *Cluster I* (29 patients) – the severely impacted patient featured highest levels of depression and migraine-related disability. *Cluster II* (28 patients) – the minimally impacted patient exhibited highest levels of self-efficacy and exercise. *Cluster III* (43 patients) – the moderately impacted patient showed features ranging between Cluster I and II. The first 5 principal components (PC) of the PCA explained 65% of variability. The first PC (eigenvalue 4.2) showed one major pattern of clinical features positively loaded by migraine-related disability, depression, poor sleep quality, somatic symptoms, post-traumatic stress disorder, being overweight and negatively loaded by pain self-efficacy and exercise levels. CM patients can be classified into three naturally-occurring clusters. Patients with high self-efficacy and exercise levels had lower migraine-related disability, depression, sleep quality, and somatic symptoms. These results may ultimately inform different management strategies.

## Introduction

Chronic migraine (CM) is a disabling, underdiagnosed and undertreated disorder afflicting about 1–2% of the general population^1^ and 9% of migraine sufferers^2^. By virtue of being a heterogenous condition with varying degree of symptomatology, comorbidities, and disability^3,4^, CM clinical semiology presents significant challenge in diagnosis, management and clinical trials^2,4^. Identifying clinically appropriate as well as naturally occurring CM clusters may help in better understanding different CM phenotypes. Classification of CM into subgroups may be useful to characterize treatment outcome determinants. According to the International Classification of Headache Disorders (ICHD-3)^5^, CM is diagnosed as headache days of 15 or more in migraine sufferers out of which 8 must be migraine. The use of 15-day cutoff is arbitrary and may not homogeneously represent all cases of CM. Some CM patients may have tolerable migraine-related disability, while others may get highly disabling migraine attacks and comorbidities despite having similar frequency of migraine days.

Unsupervised data reduction methods (e.g. clustering analysis) can be used to categorize CM cases without *a priori* knowledge on patient classification. These methods can provide evidence-based impression on multiple phenotypes of complex CM presentations beyond traditional ICHD-based diagnosis. Likewise, principal component analysis (PCA) can be applied to efficiently condense complex and multivariate diagnostic datasets for conditions as diverse as CM^6–8^. While there are published studies on exploring natural clusters in episodic migraine and other headache types^7–9^, there are no previous data reduction studies exclusively focused on exploring CM natural clusters. Our study was specifically designed with the intention to gain a deeper understanding of CM clinical phenotypes in view of the fact that CM significantly weighs on primary headache burden in clinical settings^10,11^.

In order to better characterize CM, we sought to identify clinically meaningful CM clusters within our study population by using clustering analysis and PCA.

## Results

A total of 100 CM patients completed the study. Patient characteristics are displayed in Table 1. Demographics showed that participating CM patients were middle-aged, predominantly female and mildly overweight. CM patients had high frequency of 27 monthly headache days with moderate head pain intensity, severe migraine-related disability, and a median CM duration of 7 years. More than half of the patients had medication-overuse headache (MOH) (63%). Psychological scores revealed that patients were mildly depressed with moderate level of somatic symptom severity. On average, patients had poor sleep quality, low pain self-efficacy, low exercise minutes, and regular lifestyle behavior (RLB) score of 18 out of 42.

**Table 1:** Patient Characteristics.

Characteristics	Median (IQR)
Age, years	40 (28, 53)
Female-to-Male ratio	4.56 (82/18)
BMI	26 (22, 30)
Monthly frequency of headache	27 (20, 30)
Severity of headache, NRS 0–10	6 (5, 8)
CM duration, years	7 (3, 13)
Medication-overuse headache (%)	63%
MIDAS score	80 (43, 175)
Depression score, PHQ-9	9 (6, 13)
Anxiety score, GAD-7	4 (2, 7)
Pain catastrophizing score, PCS	19 (13, 25)
Sleep quality, PSQI	9 (7, 12)
PTSD level, PC-PTSD	0 (0, 1)
Somatic symptoms, PHQ-15	12 (8, 15)
Pain self-efficacy level, PSEQ	26 (17, 34)
Exercise minutes per week	75 (15, 200)
Regular Lifestyle Behavior (RLB)	18 (10, 25)

The study patient population shared similar features to general chronic migraine population. Patients were predominantly female, young to middle-aged, mildly overweight, and having a median of 27 migraine monthly days. Patients had moderate migraine severity with a 7-year median duration of chronic migraine. The majority had medication-overuse headache and were severely disabled from recurrent migraine attacks. On average, patients suffered from some level of depression, anxiety, poor sleep quality, and somatic symptoms. Levels of self-efficacy, exercise, and regular lifestyle behavior were low on average. Abbreviations: IQR: Inter-Quartile Range, BMI: Body mass index, NRS: numerical rating scale; MIDAS^23^: Migraine Disability Assessment; PHQ-9^24^: Patient Health Questionnaire-9; GAD-7^25^: General Anxiety Disorder-7; PCS^26^: Pain Catastrophizing Scale; PSQI^27^: Pittsburgh Sleep Quality Index; PTSD: Post-Traumatic Stress Disorder assessed by PC-PTSD^28^; PHQ-15^29^: Patient Health Questionnaire-15; PSEQ^30^: Pain Self-Efficacy Questionnaire.

**Figure 1:**
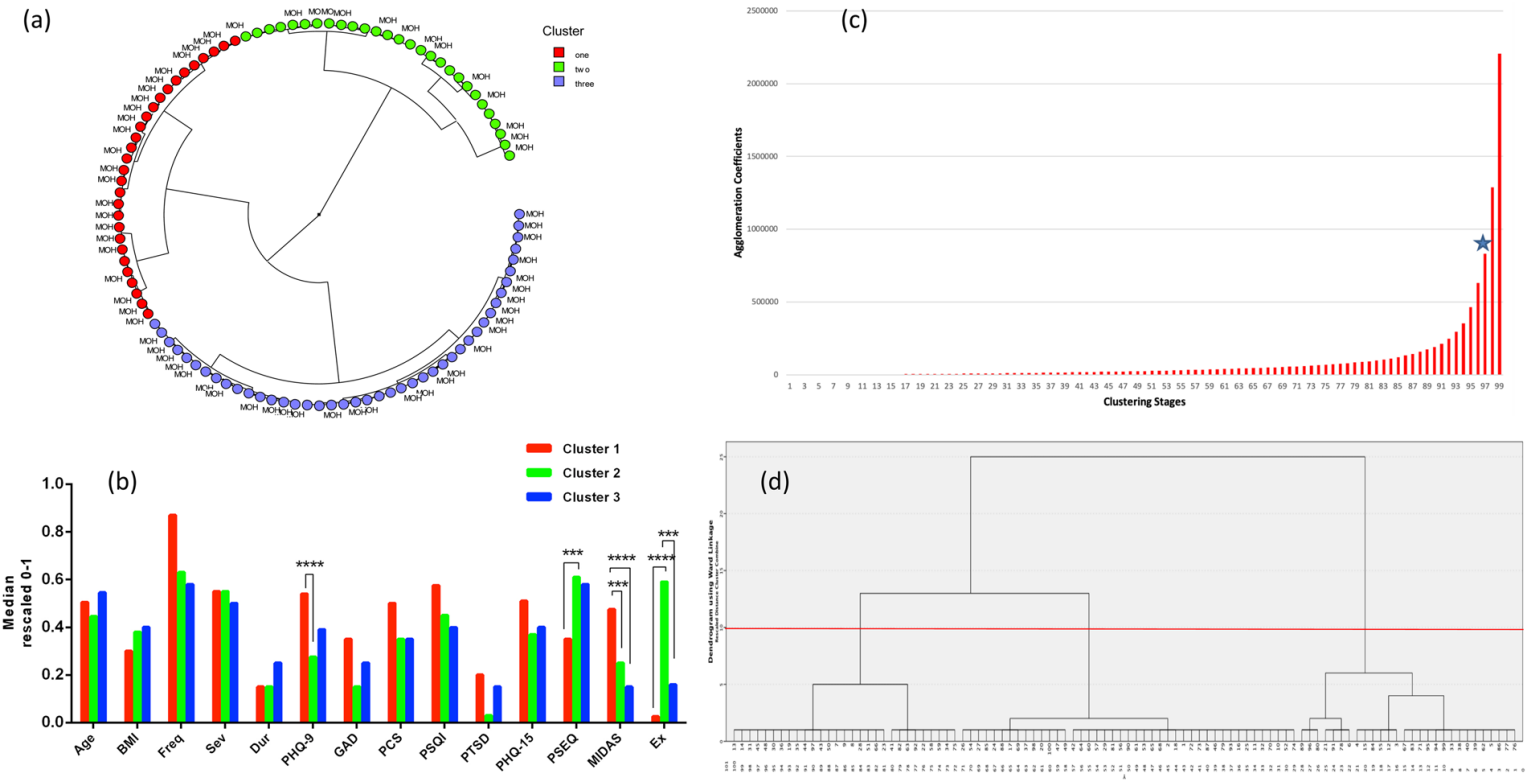
Hierarchical agglomerative clustering results. (**a)** Radial dendrogram. Hierarchical agglomerative clustering resulted in determining 3 major clusters of chronic migraine patients based on their migraine-related phenotype and comorbidities. Cluster I (red; 29 patients) – the severely impacted patient featured higher levels of depression and migraine-related disability. Cluster II (green; 28 patients) – the minimally impacted patient exhibited higher levels of pain self-efficacy and exercise. Cluster III (blue; 43 patients) – the moderately impacted patient showed most features ranging midway between Cluster I and II. CM patients with MOH are displayed next to each color. Cluster I CM patients (the severely impacted) were 4 times higher odds of having MOH compared to Cluster II CM (the minimally impacted) (*p* = 0.02). (**b**) Inter-median comparison of clinical variables among the 3 clusters. This graph shows the comparison among the median centroids of each cluster across the 14 clinical phenotype variables. Significant differences in levels of depression, pain self-efficacy, migraine-related disability, and exercise were found between Cluster I (red; the severely impacted) compared to Cluster II (green; the minimally impacted). Statistically significant difference between median centroids of all 14 variables among the three clusters was examined using Kruskal-Wallis and Dunn’s post-hoc test. Bonferroni correction was used to adjust for multiple testing by dividing *p* value of 0.05 to 14, and using new *p* value of 0.0036 as significance threshold. Significant inter-median differences with *p* value < 0.0036 are displayed. Statistically significant results are shown with asterisks as ***p < 0.001, ****p < 0.0001. Abbreviations: BMI for body mass index; Freq for frequency of headache days; Sev for severity of headache; Dur for duration of chronic migraine in years; PHQ-9^24^ instrument for assessing depression; GAD-7^25^ for assessing anxiety; PCS^26^ instrument for assessing pain catastrophizing; PSQI^27^ for assessing sleep quality; PTSD for post-traumatic stress duration assessed by PC-PTSD^28^; PHQ-15^29^ for assessing level of somatic symptoms; PSEQ^30^ for pain self-efficacy questionnaire; MIDAS^23^ for migraine disability assessment; Ex for weekly exercise minutes. The scores were all rescaled from 0–1 as shown on “y” axis. (**c**) Agglomeration coefficients schedule. The first large increase between two consecutive agglomeration coefficients is indicated by blue star at stage 97, eliminating stages 98 and 99 with resultant 3 clusters as shown in. **(d**) Dendrogram with red line indicating optimal stopping point of clustering. The red line crosses 3 vertical  lines corresponding to 3 clusters. The last 2 horizontal  lines represent the last 2 agglomeration stages (stages 98 and 99). Agglomeration coefficients schedule is shown in (**c**).

Based on visualization of the radial dendrogram (Fig. 1a), three major clusters were identified. *Cluster I* (29 patients) – the severely impacted patient featured higher levels of depression and migraine-related disability. *Cluster II* (28 patients) – the minimally impacted patient exhibited higher levels of pain self-efficacy and exercise. *Cluster III* (43 patients) – the moderately impacted patient showed most features ranging midway between Cluster I and II. Cluster I CM patients (the severely impacted) had 4 times higher odds of having MOH compared to Cluster II CM (the minimally impacted) patients (*p* = 0.02, 95% CI 1.2–12.3). Inter-median comparison (Fig. 1b) showed statistically significant differences between Cluster I (the severely impacted) and Cluster II (the minimally impacted) among the following variables (Bonferroni-adjusted to 14 variables *p* < 0.0036): depression, migraine-related disability, pain self-efficacy, exercise minutes. Similarly, the scree plot using agglomeration schedule coefficients and clustering stages indicated stage 97 to be optimal stopping point of clustering – eliminating the last 2 stages (98 and 99) and resulting in 3 distinct clusters (Fig. 1c,d). Agglomeration schedule results are available online at Supplementary Tables 3 and 4.

**Figure 2:**
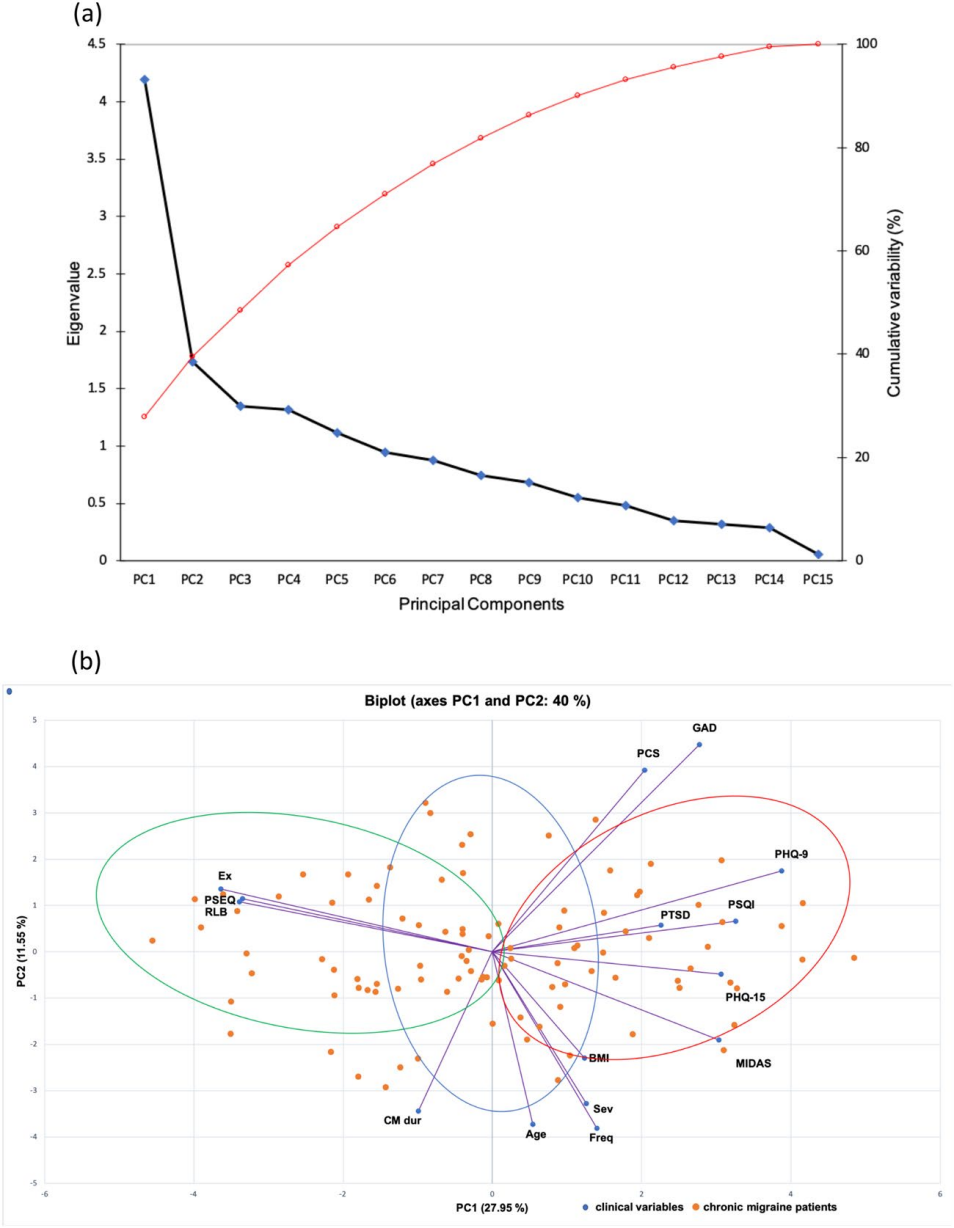
Results from PCA. (**a**) Scree plot for PCA. The first five principal components (PCs) were found to have eigenvalues greater than 1 and explained 65% of the cumulative variability (red line) within the CM phenotype dataset. The steep part of the slope (blue line) was made by the first two PCs which were used to construct the biplot in (**b**). PCs are displayed on the ‘x’ axis. The right ‘y’ axis shows the percentage of cumulative variability explained by each variable (red line). The left ‘y’ axis shows the eigenvalues for each PC. (**b**) Biplot of PCA clinical variables and chronic migraine patients. Biplot of clinical variables and participants showed differential aggregation of the 3 clusters identified on hierarchical agglomerative clustering. Ellipses show 95% confidence interval of clusters aggregation. Cluster I CM patients (red ellipse; the severely impacted) aggregated around higher migraine disability while Cluster II (green ellipse; the minimally impacted) assembled around higher self-efficacy and exercise levels. Cluster III (blue ellipse; the moderately impacted) patients were scattered between Clusters I and II.

For PCA, the Kayser-Meyer-Olkin (KMO) value of 0.70 indicated adequacy of sampling. Bartlett’s sphericity test (*p* < 0.0001) showed no identity matrix within the PCA signifying the dataset’s suitability for detection of principal components (PC). The first five PCs were found to have eigenvalues greater than 1 and explained 65% of the variability within the CM phenotype dataset (Fig. 2a). The first 2 PCs (i.e. PC1 and PC2) made the steep part of the scree plot – hence, PC1 (eigenvalue 4.2) and PC2 (eigenvalue 1.7) were selected to plot the PCA biplot of clinical variables and patients’ distribution across the PCs (Fig. 2b). The PCA biplot revealed one major pattern of clinical features positively loaded by migraine-related disability, depression, poor sleep quality, somatic symptoms, post-traumatic stress disorder, being overweight and negatively loaded by pain self-efficacy, exercise, and RLB levels. This indicated the inverse relationship between positively and negatively loaded variables. In addition, the biplot (Fig. 2b) assessment displayed the 95% confidence interval for the distinct 3 clusters identified in the hierarchical agglomerative clustering (HAC); Cluster I CM patients (red; the severely impacted) aggregated around higher migraine disability and associated psychosomatic comorbidities  while Cluster II (green; the minimally impacted) assembled around higher self-efficacy, exercise and RLB levels. Cluster III (blue; the moderately impacted) patients were scattered between Clusters I and II.

Correlogram (see Supplementary Fig. S1) of association among the 14 clinical variables showed statistically significant association (Bonferroni-adjusted to 91 association tests *p* < 0.0005) between migraine frequency and migraine-related disability. Furthermore, there was positive association between depression and anxiety, pain catastrophizing, poor sleep quality, PTSD, somatic symptoms, migraine-related disability. Pain self-efficacy and exercise level exhibited inverse relationship to depression, poor sleep quality. Pain self-efficacy and exercise level had positive association. Anxiety showed positive association with pain catastrophizing and sleep quality. Poor sleep quality displayed positive association with somatic symptoms level. Heatmap results (see Supplementary Fig. S2) corroborated with correlogram by showing that features such as increased pain self-efficacy and exercise were associated with lower migraine burden and psychological comorbidities.

The results after excluding cases with missing data revealed findings similar to results in which missing data were replaced by medians. PCA showed one major pattern of clinical features positively loaded by migraine-related disability, depression, poor sleep quality, somatic symptoms, post-traumatic stress disorder, and negatively loaded by pain self-efficacy, exercise, and RLB levels (Supplementary Fig. S3). Additionally, the clustering analysis after excluding cases with missing data showed 3 major clusters (Supplementary Figs. S4 and S5) similar to our results in which missing data were replaced by medians: severely impacted cluster featuring low levels of self-efficacy and exercise with high levels of psychological comorbidities, minimally impacted cluster featuring high levels of self-efficacy and exercise, and moderately impacted with features ranging between the severely and minimally impacted clusters. The complete dataset is available in Supplementary Table 2.

## Discussion

This study proved that CM can be classified into three naturally occurring clusters using clinical datasets. The three clusters were found to be clinically meaningful, for example Cluster II (the minimally impacted) with higher pain self-efficacy, exercise, and regular lifestyle behavior (RLB) levels corresponded to lower migraine-related disability and comorbidities compared to Cluster I (the severely impacted). Additionally, the minimally impacted Cluster II CM patients had 4 times lower odds of having comorbid MOH compared to the severely impacted Cluster I CM patients. Inverse association between pain self-efficacy, exercise, RLB on one hand, and migraine disability, comorbidities on the other indicates that the impact of self-efficacy and exercise may stem not only from reducing migraine pain behavior but also from neuromodulation.

Our results support the social-cognitive theory proposed by other authors to explain bidirectional mechanism of self-efficacy and exercise being coupled with reduced migraine burden and comorbidities^12^. That self-efficacy, RLB, and exercise levels positively correlated to each other while being inversely related to migraine disability and comorbidities corroborates the link between lifestyle behaviors and migraine self-management^13,14^. A randomized controlled trial and other interventional as well as observational studies have shown the efficacy of regular lifestyle behaviors such as regular exercise^13,14^, regular sleep^15^, regular water intake^16^, and avoiding skipped mealtimes^17^ in reducing migraine attacks. Improving self-efficacy and exercise is thought to have several advantages in migraine management by improving internal locus of control^12,13^, self-management^12,13^, outcome expectancy^18^, affect and mood state^19^, and addressing psychopathology in depression and anxiety^12^. The median weekly exercise of 210 minutes found in Cluster II (the minimally impacted) indicates that a 30-minute daily exercise was associated with reduced migraine attacks in CM. That the three clusters featured similar migraine severity and frequency but differing disability, self-efficacy and depression levels reflects CM heterogeneity.

The agreement between the clusters which emerged from HAC and the dominant PCA pattern validates our findings. HAC algorithm with a bottom-up approach separated clinically appropriate clusters within the study population. Determining outcome of CM patients merely on the basis of change in headache days may not consistently result in optimum patient satisfaction. Utilization of multivariate CM datasets provides deeper insight leading the way to precision medicine in headache medicine. Combination of *heterogenous* CM patients under the umbrella of ‘chronic migraine’ may account for varying degrees of treatment response in clinical trials. Our findings can be used to identify distinct naturally occurring clusters of CM patients who benefit most from targeted interventions for behavioral change e.g. social-cognitive or learning theory^20^ to improve self-efficacy and exercising^21^. Moreover, our cluster identification can be applied to discover biomarkers (e.g. genes, proteins) linked to a specific cluster. Our study clearly showed significant heterogeneity in patient characteristics and comorbidities despite all patients having been diagnosed as CM. Improved recognition of such heterogeneity may lead to more potent treatment by personalizing headache care to better fit CM patient profiles. Multidimensionality of CM clinical profiles can be reduced using the approach in our study.

Our study’s limitations are inherent to cross-sectional design which necessitate validation of association results. Prospective studies are required to further establish temporal relationship between correlated clinical variables e.g. that self-efficacy leads to lower disability in CM. Multi-center and larger sample-sized community-based studies will be needed to fully endorse our results for generalizability beyond tertiary headache centers. That being said, our KMO of 0.70 and significant Bartlett’s sphericity are indicators of the suitability of our multidimensional dataset for reduction. Our study population shared similar features to target population of CM^2–4^ indicating some degree of representativeness i.e. predominantly female, middle-aged, mildly overweight, high migraine-related disability, and psychological comorbidities (Table 1). However, by virtue of being from a tertiary headache clinical center involving patients who have undergone several referrals and treatment failures, our study source population may not fully represent the general CM population in the community.

Based on results from these unsupervised methods, we are developing supervised learning algorithm to generate a predictive model for CM classification. Development of such models may help in predicting treatment response and creating distinct baseline patient classification for clinical trials. Baseline classification of CM patients will be crucial in identifying non-responders, responders, and super-responders. Our ongoing research includes determining links between these CM clusters, biological markers, and neuroimaging correlates in a longitudinal study design.

## Methods

### Study design and patients recruitment

This was a cross-sectional clinical study with the following inclusion criteria: CM patients who were 18 years and older, CM diagnosis made by headache specialist according to International Classification of Headache Disorders 3-beta (ICHD 3-beta)^22^ criteria, minimum CM duration of 1 year, and ability to speak and write in English. Patients were allowed to be on their usual care and medications. Exclusion criteria were children under age 18, inability to speak and write in English and secondary headaches other than comorbid medication-overuse headache (MOH). Patients were recruited from the Stanford Headache Clinic between January 2015–May 2019.

### Phenotyping and assessing comorbidities

#### Migraine-related questionnaires

All CM patients completed online self-administered questionnaires about their demographic information, duration of CM, headache features during the previous 3 months involving monthly frequency of headache days, headache severity on numeric rating scale of 0 to 10, headache medication use, and headache-related disability measured using Migraine Disability Assessment (MIDAS)^23^. Additionally, CM patients provided information on lifetime duration of migraine.

#### Psychometric questionnaires

In order to assess for comorbid psychological and behavioral conditions, CM patients completed the following questionnaires: Patient Health Questionnaire-9 (PHQ-9)^24^ for depression, Generalized Anxiety Disorder-7 (GAD-7)^25^ for anxiety, Pain Catastrophizing Scale (PCS)^26^ to assess pain catastrophizing, Pittsburgh Sleep Quality Index (PSQI)^27^ for sleep quality, Primary Care Post-Traumatic Stress Disorder (PC-PTSD)^28^ to assess for PTSD, Patient Health Questionnaire-15 (PHQ-15)^29^ for somatic symptoms, and Pain Self-Efficacy Questionnaire (PSEQ)^30^ to examine patients’ confidence in performing daily activities despite head pain. Exercise level was measured using self-administered online questionnaire asking for weekly aerobic exercise minutes. Regular lifestyle behavior (RLB)^31^ was scored by assigning 7 points each to regular wake time and regular sleep time, 14 points to regular mealtime, and weekly exercise minutes scored as 0–14 points graded at 30-minute interval ranging from 0 to 420 minutes or above. A complete rubric scoring system for RLB is provided in Supplementary Table 1.

### Statistical analysis

The sample size was based on the available data. No statistical power calculation was conducted prior to the study. This is the primary analysis of these data. Descriptive statistics were used to analyze demographic data, migraine-related clinical features, and questionnaire scores. Clustering analysis was performed using Ward’s agglomeration method of hierarchical agglomerative clustering (HAC) with Squared Euclidean distance metric as measure of dissimilarity. Results from clustering analysis were utilized to identify natural clusters of CM patients using 14 migraine-related clinical variables (i.e. age, body mass index or BMI, monthly headache frequency, average headache severity, CM duration, depression, anxiety, pain catastrophizing, sleep quality, PTSD, somatic symptoms, migraine-related disability, pain self-efficacy, and exercise level). A dendrogram representing the patients grouped in clusters was prepared to visualize the HAC clustering process. A scree plot of agglomeration schedule coefficients by clustering stage was prepared. Optimum cutoff for natural clustering was selected by using the “elbow method” from the scree plot. The “elbow method” incorporates the stage with steep-to-shallow slope change to define the optimal cutoff^32^. In addition, the dendrogram was visualized to aid in optimal cutoff selection. In order to evaluate whether the final clustering satisfactorily differentiates the dataset, the cluster centroids were examined by comparing the medians of the 14 variables across the clusters using Kruskal-Wallis test with Dunn’s post-hoc.

Furthermore, principal component analysis (PCA) was used to demonstrate the accuracy of our finding with HAC and to condense the 14 clinical variables to those explaining the largest variation. Considering the different measurement scales used for the clinical variables, PCA was built on Spearman correlation matrix. Kaiser-Meyer-Olkin (KMO) measure was carried out for assessing sampling adequacy. Bartlett’s sphericity test was used to determine whether the covariance matrix contained an identity matrix. The Kaiser criterion was applied to retain principal components (PCs) with eigenvalue greater than 1^33^. A scree plot of principal components by eigenvalues was graphed to examine whether the PCA is applicable for our dataset^34^. The “elbow method” was used to capture the PCs explaining the largest variability^34^. A PCA biplot was utilized to describe the relationship between the participants and variable loadings.

Association analysis between the 14 clinical features was conducted using Spearman’s *ρ* in a correlogram. Two-tailed *p* value of 0.05 was considered as significant threshold for association analysis with Bonferroni correction applied to correct for multiple testing. Missing data was replaced by median in 79 of the datapoints across 10 of the 100 cases included in HAC and PCA analysis. In total, there was 5.5% of missing data (88 out of 1600 datapoints from 16 cases, including MOH data).

Statistical analyses were done using Statistical Package for Social Sciences (version 21.0; SPSS Inc, Chicago IL), BioNumerics version 7.6 created by Applied Maths NV; Available from http://www.applied-maths.com, and XLSTAT 2019 (Addinsoft).

### Standard protocol approvals, registrations, and patient consents

All participants signed informed consent prior to study procedures. The study is approved by the Stanford University Institutional Review Board (IRB-30785) and has therefore been performed in accordance with the ethical standards laid down in the 1964 Declaration of Helsinki and its later amendments.

## Supplementary information

Supplementary File.

## Data Availability

The datasets generated during and/or analysed during the current study are available in Supplementary Files Online.
