# Supplementary material for: Exploring Natural Clusters of Chronic Migraine Phenotypes: A Cross-Sectional Clinical Study
Source: Sci Rep. 2020 Feb 18;10:2804. doi: 10.1038/s41598-020-59738-1 (PMC7028739; doi:10.1038/s41598-020-59738-1)
Supplement: Supplementary file 1 — Supplementary File. [file 41598_2020_59738_MOESM1_ESM.pdf]

Title: Exploring Natural Clusters of Chronic Migraine Phenotypes: A Cross-Sectional Clinical Study

Authors: Yohannes W. Woldeamanuel, MD; Bharati M. Sanjanwala, MSc; Addie M. Peretz, MD; Robert P. Cowan, MD

Supplementary Figure S1

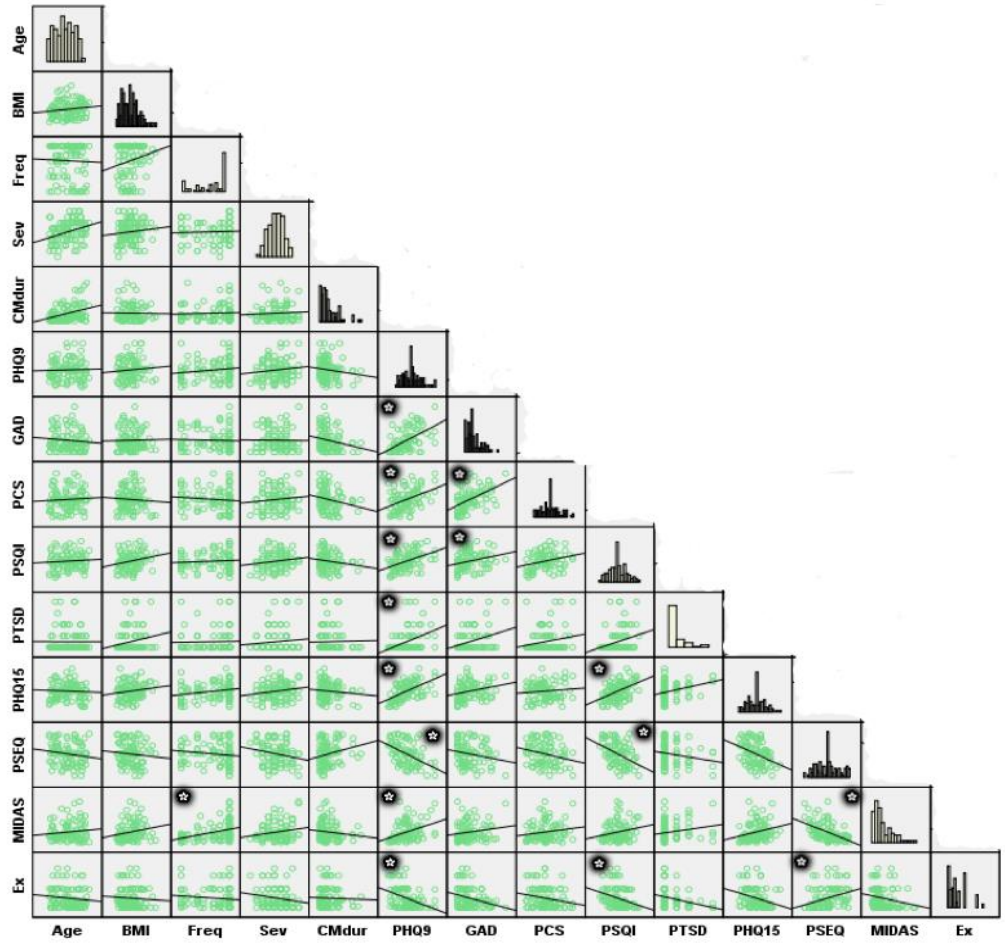

Supplementary Figure S1. Correlogram displaying association matrix among the 14 variables.

Correlogram of association among the 14 clinical variables showed statistically significant association between variables indicating migraine burden (e.g. migraine frequency, disability) and psychological comorbidities (e.g. depression). There was inverse relationship between psychological comorbidities and behaviors such as pain self-efficacy and exercise level.

Statistically significant associations are marked with asterisks. Spearman's ρ was used for determining association statistics, and significance level was adjusted using Bonferroni method for multiple testing by dividing 0.05 to 91 associations. A new significance threshold of $p < 0.0005$ was used. Distribution histograms are shown along diagonal space. Abbreviations are explained in legend of Figure 1b.

Supplementary Figure S2

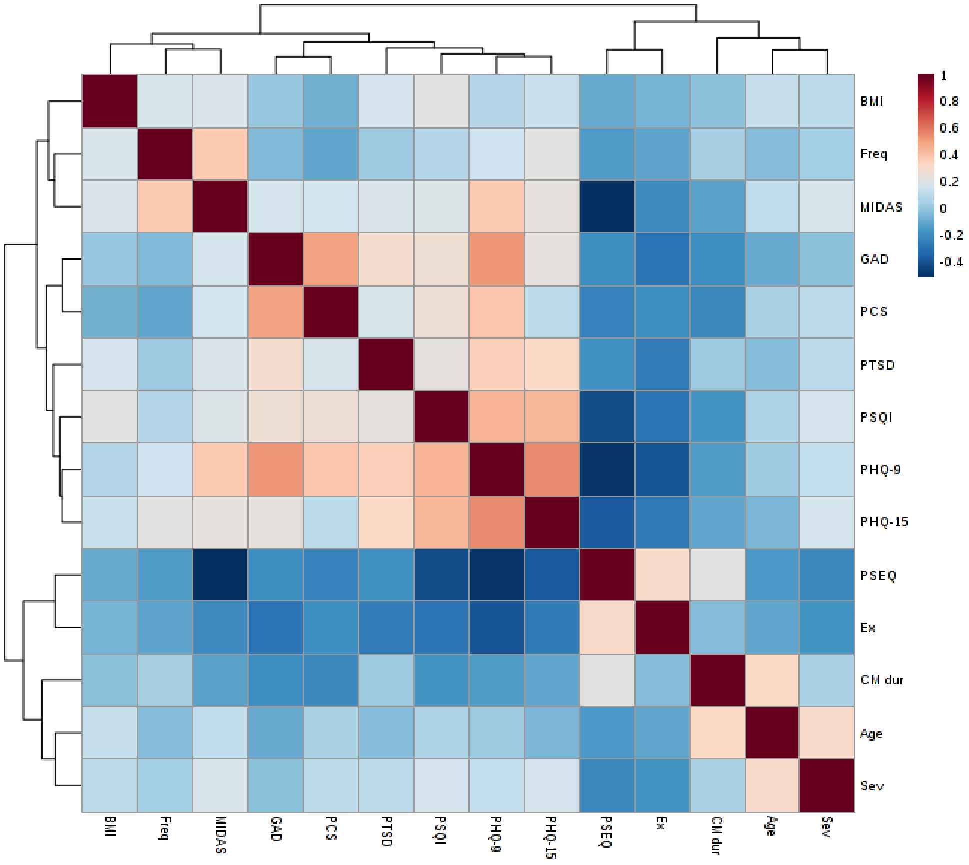

Supplementary Figure S2. Heatmap showing clustering of clinical variables.

The heatmap displays clustering of clinical variables that are highly correlated and anticorrelated using the color index. Variables such as exercise and pain self-efficacy assembled into one subgroup. Migraine-related psychological comorbidities fell into another subgroup. The agglomeration shows that chronic migraine patients with lower exercise and pain self-efficacy levels tend to have higher migraine disability and psychological comorbidities. Clustering was based on hierarchical agglomerative clustering. Spearman's ρ was used for determining

correlations. Red boxes indicate increased correlation while blue boxes indicate inverse correlation.

Supplementary Fig. S3

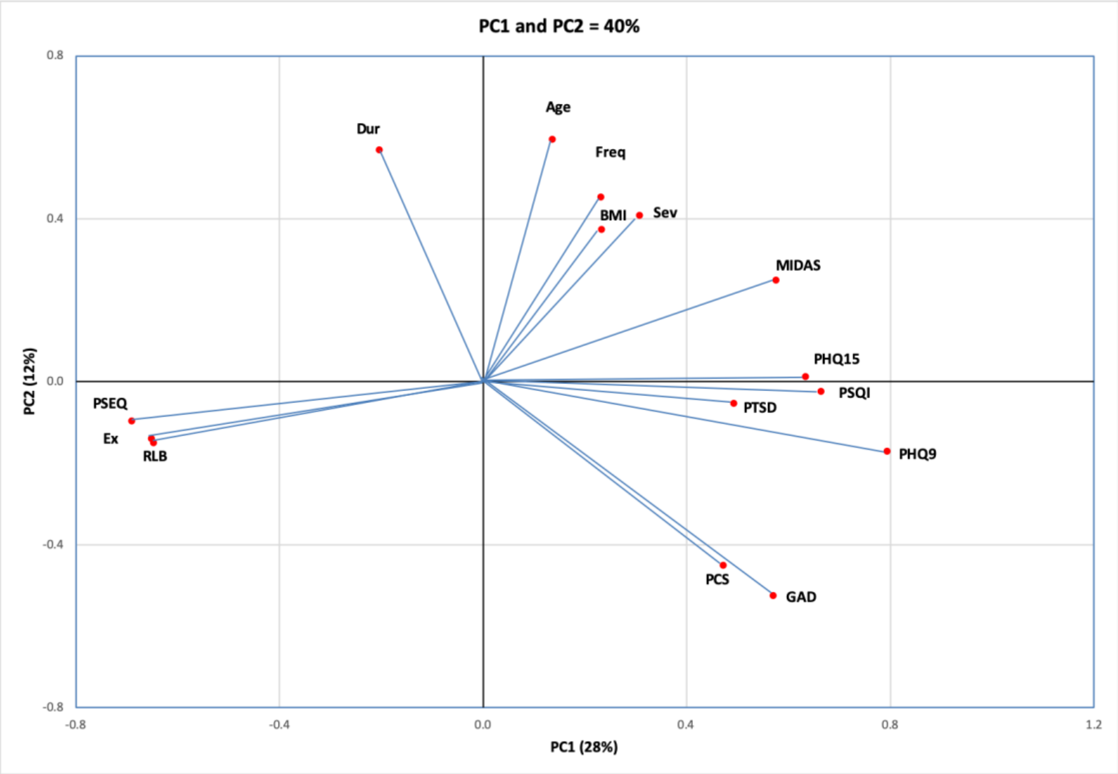

Supplementary Figure S3. Principal components analysis (PCA) after excluding cases with missing datapoints. PCA showed one major pattern of clinical features positively loaded by migraine-related disability, depression, poor sleep quality, somatic symptoms, post-traumatic stress disorder, and negatively loaded by pain self-efficacy, exercise, and RLB levels. These results were similar to the PCA with missing datapoints replaced by medians (Figure 2b).

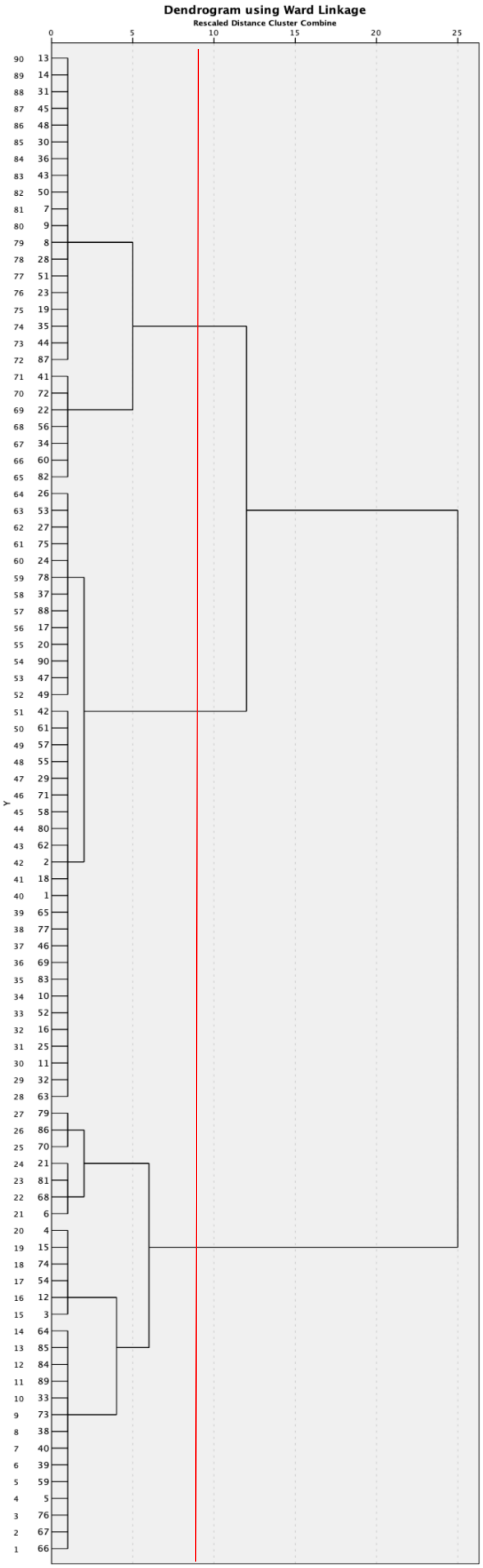

Supplementary Figure S4. Dendrogram with red line indicating optimal stopping point of clustering after excluding cases with missing datapoints. The red line crosses 3 horizontal lines corresponding to 3 clusters. The last 2 vertical lines represent the last 2 agglomeration stages (stages 88 and 89). Agglomeration coefficients schedule is shown in Supplementary Fig. S5.

Supplementary Figure S5

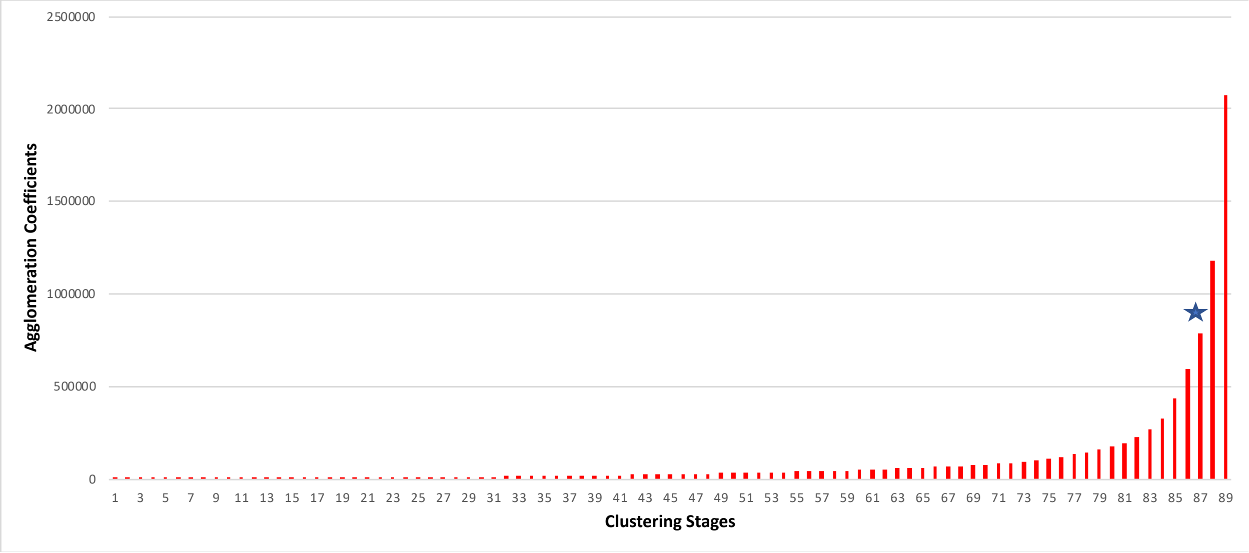

Supplementary Figure S5. Agglomeration schedule coefficients after excluding cases with missing datapoints. The first large increase between two consecutive agglomeration coefficients is indicated by blue star at stage 87, eliminating stages 88 and 89 with resultant 3 clusters as shown in Supplementary Fig. S4.

Supplementary Table 1: Rubric scoring for regular lifestyle behavior (RLB) questionnaire.

	**Wake up time:** Regular/Irregular (score)	**Sleep time:** Regular/Irregular (score)	**Mealtimes:** Regular/Irregular (score)	**Exercise:** weekly minutes (score)	**Total score**
	Regular (7)	Regular (7)	Regular (14)	≥ 390 (14)	42
	Irregular (0)	Regular (7)	Regular (14)	≥ 390 (14)	35
	Regular (7)	Irregular (0)	Regular (14)	≥ 390 (14)	35
	Regular (7)	Regular (7)	Irregular (0)	≥ 390 (14)	28
	Regular (7)	Regular (7)	Regular (14)	360 – 389 (13)	41
	Irregular (0)	Regular (7)	Regular (14)	360 – 389 (13)	34
	Regular (7)	Irregular (0)	Regular (14)	360 – 389 (13)	34
	Regular (7)	Regular (7)	Irregular (0)	360 – 389 (13)	27
	Regular (7)	Regular (7)	Regular (14)	330 – 359 (12)	40
	Irregular (0)	Regular (7)	Regular (14)	330 – 359 (12)	33
	Regular (7)	Irregular (0)	Regular (14)	330 – 359 (12)	33
	Regular (7)	Regular (7)	Irregular (0)	330 – 359 (12)	26
	Regular (7)	Regular (7)	Regular (14)	300 – 329 (11)	39
	Irregular (0)	Regular (7)	Regular (14)	300 – 329 (11)	32
	Regular (7)	Irregular (0)	Regular (14)	300 – 329 (11)	32
	Regular (7)	Regular (7)	Irregular (0)	300 – 329 (11)	25
	Regular (7)	Regular (7)	Regular (14)	270 – 299 (10)	38
	Irregular (0)	Regular (7)	Regular (14)	270 – 299 (10)	31
	Regular (7)	Irregular (0)	Regular (14)	270 – 299 (10)	31
	Regular (7)	Regular (7)	Irregular (0)	270 – 299 (10)	24
	Regular (7)	Regular (7)	Regular (14)	240 – 269 (9)	37
	Irregular (0)	Regular (7)	Regular (14)	240 – 269 (9)	30
	Regular (7)	Irregular (0)	Regular (14)	240 – 269 (9)	30
	Regular (7)	Regular (7)	Irregular (0)	240 – 269 (9)	23
	Regular (7)	Regular (7)	Regular (14)	210 – 239 (8)	36
	Irregular (0)	Regular (7)	Regular (14)	210 – 239 (8)	29
	Regular (7)	Irregular (0)	Regular (14)	210 – 239 (8)	29
	Regular (7)	Regular (7)	Irregular (0)	210 – 239 (8)	22
	Regular (7)	Regular (7)	Regular (14)	180 – 209 (7)	35
	Irregular (0)	Regular (7)	Regular (14)	180 – 209 (7)	28
	Regular (7)	Irregular (0)	Regular (14)	180 – 209 (7)	28
	Regular (7)	Regular (7)	Irregular (0)	180 – 209 (7)	21
	Regular (7)	Regular (7)	Regular (14)	150 – 179 (6)	34
	Irregular (0)	Regular (7)	Regular (14)	150 – 179 (6)	27
	Regular (7)	Irregular (0)	Regular (14)	150 – 179 (6)	27
	Regular (7)	Regular (7)	Irregular (0)	150 – 179 (6)	20
	Regular (7)	Regular (7)	Regular (14)	120 – 149 (5)	33
	Irregular (0)	Regular (7)	Regular (14)	120 – 149 (5)	26

	Regular (7)	Irregular (0)	Regular (14)	120 – 149 (5)	26
	Regular (7)	Regular (7)	Irregular (0)	120 – 149 (5)	19
	Regular (7)	Regular (7)	Regular (14)	90 – 119 (4)	32
	Irregular (0)	Regular (7)	Regular (14)	90 – 119 (4)	25
	Regular (7)	Irregular (0)	Regular (14)	90 – 119 (4)	25
	Regular (7)	Regular (7)	Irregular (0)	90 – 119 (4)	18
	Regular (7)	Regular (7)	Regular (14)	60 – 89 (3)	31
	Irregular (0)	Regular (7)	Regular (14)	60 – 89 (3)	24
	Regular (7)	Irregular (0)	Regular (14)	60 – 89 (3)	24
	Regular (7)	Regular (7)	Irregular (0)	60 – 89 (3)	17
	Regular (7)	Regular (7)	Regular (14)	30 – 59 (2)	30
	Irregular (0)	Regular (7)	Regular (14)	30 – 59 (2)	23
	Regular (7)	Irregular (0)	Regular (14)	30 – 59 (2)	23
	Regular (7)	Regular (7)	Irregular (0)	30 – 59 (2)	16
	Regular (7)	Regular (7)	Regular (14)	1 – 29 (1)	29
	Irregular (0)	Regular (7)	Regular (14)	1 – 29 (1)	22
	Regular (7)	Irregular (0)	Regular (14)	1 – 29 (1)	22
	Regular (7)	Regular (7)	Irregular (0)	1 – 29 (1)	15
	Regular (7)	Regular (7)	Regular (14)	0 (0)	28
	Irregular (0)	Regular (7)	Regular (14)	0 (0)	21
	Regular (7)	Irregular (0)	Regular (14)	0 (0)	21
	Regular (7)	Regular (7)	Irregular (0)	0 (0)	14
	Irregular (0)	Irregular (0)	Regular (14)	≥ 390 (14)	28
	Irregular (0)	Irregular (0)	Irregular (0)	≥ 390 (14)	14
	Regular (7)	Irregular (0)	Irregular (0)	≥ 390 (14)	21
	Irregular (0)	Irregular (0)	Regular (14)	360 – 389 (13)	27
	Irregular (0)	Irregular (0)	Irregular (0)	360 – 389 (13)	13
	Regular (7)	Irregular (0)	Irregular (0)	360 – 389 (13)	20
	Irregular (0)	Irregular (0)	Regular (14)	330 – 359 (12)	26
	Irregular (0)	Irregular (0)	Irregular (0)	330 – 359 (12)	12
	Regular (7)	Irregular (0)	Irregular (0)	330 – 359 (12)	19
	Irregular (0)	Irregular (0)	Regular (14)	300 – 329 (11)	25
	Irregular (0)	Irregular (0)	Irregular (0)	300 – 329 (11)	11
	Regular (7)	Irregular (0)	Irregular (0)	300 – 329 (11)	18
	Irregular (0)	Irregular (0)	Regular (14)	270 – 299 (10)	24
	Irregular (0)	Irregular (0)	Irregular (0)	270 – 299 (10)	10
	Regular (7)	Irregular (0)	Irregular (0)	270 – 299 (10)	17
	Irregular (0)	Irregular (0)	Regular (14)	240 – 269 (9)	23
	Irregular (0)	Irregular (0)	Irregular (0)	240 – 269 (9)	9
	Regular (7)	Irregular (0)	Irregular (0)	240 – 269 (9)	16
	Irregular (0)	Irregular (0)	Regular (14)	210 – 239 (8)	22
	Irregular (0)	Irregular (0)	Irregular (0)	210 – 239 (8)	8
	Regular (7)	Irregular (0)	Irregular (0)	210 – 239 (8)	15
	Irregular (0)	Irregular (0)	Regular (14)	180 – 209 (7)	21
	Irregular (0)	Irregular (0)	Irregular (0)	180 – 209 (7)	7

	Regular (7)	Irregular (0)	Irregular (0)	180 – 209 (7)	14
	Irregular (0)	Irregular (0)	Regular (14)	150 – 179 (6)	20
	Irregular (0)	Irregular (0)	Irregular (0)	150 – 179 (6)	6
	Regular (7)	Irregular (0)	Irregular (0)	150 – 179 (6)	13
	Irregular (0)	Irregular (0)	Regular (14)	120 – 149 (5)	19
	Irregular (0)	Irregular (0)	Irregular (0)	120 – 149 (5)	5
	Regular (7)	Irregular (0)	Irregular (0)	120 – 149 (5)	12
	Irregular (0)	Irregular (0)	Regular (14)	90 – 119 (4)	18
	Irregular (0)	Irregular (0)	Irregular (0)	90 – 119 (4)	4
	Regular (7)	Irregular (0)	Irregular (0)	90 – 119 (4)	11
	Irregular (0)	Irregular (0)	Regular (14)	60 – 89 (3)	17
	Irregular (0)	Irregular (0)	Irregular (0)	60 – 89 (3)	3
	Regular (7)	Irregular (0)	Irregular (0)	60 – 89 (3)	10
	Irregular (0)	Irregular (0)	Regular (14)	30 – 59 (2)	16
	Irregular (0)	Irregular (0)	Irregular (0)	30 – 59 (2)	2
	Regular (7)	Irregular (0)	Irregular (0)	30 – 59 (2)	9
	Irregular (0)	Irregular (0)	Regular (14)	1 – 29 (1)	15
	Irregular (0)	Irregular (0)	Irregular (0)	1 – 29 (1)	1
	Regular (7)	Irregular (0)	Irregular (0)	1 – 29 (1)	8
	Irregular (0)	Irregular (0)	Regular (14)	0 (0)	14
	Irregular (0)	Irregular (0)	Irregular (0)	0 (0)	0
	Regular (7)	Irregular (0)	Irregular (0)	0 (0)	7

Supplementary Table 2. Phenotype dataset for each participating patient. Datapoints in bold represent median values that replaced missing values: 79 datapoints for 10 patients across 15 variables. MOH was not included in HAC and PCA analysis; 6 additional cases had missing MOH data. N/A (not available).

Age	BMI	Freq	Sev	CM dur	PHQ-9	GAD	PCS	PSQI	PTSD	PHQ-15	PSEQ	MIDAS	Ex	RLB	MOH
23	21.5	20	7	1	15	10	34	4	0	11	44	18	75	19	no
28	26	20	5	7.5	18	12	25	12	3	9	32	45	75	19	yes
28	26	15	6	7.5	4	2	19	6	0	7	34	65	430	33	yes
39	22.3	25	6	4	1	2	10	8	0	5	45	25	350	31	no
21	28	15	7	8	5	10	25	12	0	6	34	78	200	25	no
33	33.3	30	6	1	10	2	14	9	0	11	6	372	210	27	no
57	33.7	30	8	3	16	4	15	11	2	15	17	159	0	9	**N/A**
24	23.2	30	7	7	8	4	39	7	0	19	27	110	0	9	no
51	29.5	30	4	14	11	2	6	6	0	15	12	145	0	9	yes
39	40.3	29	5	4	3	4	2	5	0	12	35	100	120	23	no
18	18.3	20	3	3	3	0	6	3	0	4	47	65	120	23	no
58	21.4	15	4	7	0	0	37	10	0	3	35	0	350	31	yes
38	27.5	27	4	7.5	15	14	31	10	0	8	11	188	15	11	yes
38	27.5	27	4	7.5	15	14	28	10	0	8	11	188	15	11	yes
28	19.4	27	2	7.5	2	0	2	3	0	7	38	39	350	31	no
18	25.4	15	9	1	6	3	13	9	0	5	18	73	120	17	yes
40	19	19	6	7.5	2	12	20	6	0	6	26	5	40	13	yes
49	29.6	15	3	1	11	12	20	8	1	12	41	38	90	21	yes
51	22.9	29	6	7	11	5	21	8	1	13	21	236	75	21	no
45	42.2	28	7	5	4	1	2	13	0	16	26	24	0	3	yes
62	30.4	24	7	1	8	1	35	6	0	8	19	213	210	27	yes
57	32.1	30	5	3	20	8	24	18	1	12	2	279	0	6	no
20	26	25	4	10	14	5	15	15	0	18	28	130	40	7	yes
29	31.7	25	3	1	5	2	19	6	0	9	47	65	0	3	no
36	20.8	20	8	1	7	3	10	7	1	13	14	60	120	23	no
43	22.2	30	10	2	12	1	7	10	0	19	11	80	0	9	yes
20	22.8	30	5	3	6	2	7	11	1	12	17	59	0	2	yes
25	29.9	30	6	5	14	5	30	17	4	20	12	113	0	9	yes
54	19.3	30	10	34	9	4	19	9	0	12	26	90	75	18	**N/A**
40	36	20	8	17	10	12	7	13	1	14	43	185	45	15	yes
21	21.3	30	5	1	17	14	35	12	0	9	7	188	0	2	yes

25	27.8	30	4	11	1	0	2	2	0	6	34	44	120	23	yes
46	21.3	16	6	5	3	2	35	8	0	6	18	48	200	25	no
48	35	30	9	4	9	3	19	16	0	16	7	252	0	3	no
34	29.4	30	5	2	10	7	16	12	2	16	8	219	40	10	yes
61	27.5	30	4	40	4	0	6	5	0	3	22	180	15	14	yes
26	26.6	25	4	1	4	5	48	11	0	8	27	33	45	12	no
46	19.8	29	5	5	16	4	16	13	0	24	14	130	200	25	no
28	32.7	24	5	1	7	10	14	8	0	8	35	74	210	30	no
36	33.5	25	7	15	10	2	14	15	2	12	30	110	200	28	yes
49	28.9	30	10	5	24	20	48	10	4	20	2	430	0	12	yes
63	20.7	15	7	1	7	2	25	7	1	6	14	101	45	15	yes
53	28.6	30	8	10	12	7	45	12	1	12	10	130	0	12	yes
22	29.4	20	5	1	13	13	40	8	1	16	30	161	75	19	yes
38	19.2	25	7	3	13	5	27	16	0	18	21	170	0	3	yes
35	28	22	6	23	10	3	21	9	0	10	14	55	90	15	no
56	23.9	15	9	20	3	2	28	7	0	8	29	25	15	8	**N/A**
57	33.9	30	9	3	10	11	30	10	0	9	17	201	0	12	no
57	30.1	30	8	35	1	0	18	9	0	4	37	20	15	14	no
58	23.6	22	6	5	14	10	29	13	0	15	7	140	0	6	no
41	29.3	30	7	1	11	7	25	12	0	13	28	125	15	8	yes
41	28.7	30	6	10	17	9	38	4	0	9	24	90	120	26	no
23	21.9	20	3	7	**9**	**4**	**19**	**9**	**0**	**12**	**26**	**80**	**75**	**18**	**N/A**
34	22.4	16	6	20	10	7	14	12	2	14	17	80	15	2	yes
50	26.7	28	5	20	8	6	11	7	0	14	46	63	350	31	yes
41	25.7	30	**6**	13	**9**	**4**	**19**	**9**	**0**	12	26	80	75	18	**N/A**
63	30.3	25	6	7	13	7	17	12	1	16	27	60	45	15	no
43	24.7	30	5	5	17	13	40	17	2	19	22	282	0	9	yes
62	20.7	21	8	5	**9**	**4**	**19**	**9**	**0**	12	26	291	0	13	yes
49	22.5	15	8	19	9	13	35	6	4	17	27	86	45	18	yes
39	19.8	30	5	4	10	3	21	7	0	10	21	84	75	19	yes
28	38.4	25	7	7.5	9	6	18	11	0	17	30	65	210	24	yes
56	31.3	27	6	7.5	13	5	24	14	2	9	13	319	15	14	yes
47	27	17	9	7	17	0	11	7	0	11	15	89	45	9	yes
31	31.9	30	7	2	**9**	**4**	**19**	**9**	**0**	**12**	**26**	**80**	**75**	**18**	**N/A**
40	21.3	30	8	7	**9**	**4**	**19**	**9**	**0**	**12**	**26**	100	15	8	yes
54	20.5	15	7	5	**9**	**4**	**19**	**9**	**0**	**12**	**26**	0	200	28	yes
25	23.3	30	5	3	10	7	24	4	2	8	45	70	75	16	no
53	26	22	8	7	**9**	**4**	**19**	**9**	**0**	12	26	20	45	18	yes
26	18.6	17	4	5	13	11	26	8	0	13	38	40	120	23	no

20	19.8	22	4	5	5	3	9	14	0	7	37	15	200	22	no
45	21.3	30	8	7	9	8	13	10	0	16	26	41	75	22	yes
46	22	30	8	7	12	16	13	**9**	**0**	**12**	**26**	41	**75**	22	yes
64	24	17	7	43	**9**	**4**	**19**	**9**	**0**	**12**	**26**	**80**	120	26	yes
19	26	30	9	2	**9**	**4**	**19**	**9**	**0**	**12**	**26**	263	0	3	yes
39	32.8	15	7	1	24	4	40	14	0	22	10	80	200	28	yes
52	34.8	27	3	17	4	2	12	9	1	15	39	66	200	28	yes
23	27.9	30	7	13	9	4	19	9	0	12	26	273	210	18	**N/A**
49	19.2	30	8	24	16	0	22	8	1	14	47	60	90	24	yes
40	30.2	30	9	1	9	4	19	9	0	12	26	203	430	30	yes
59	33.3	28	8	21	24	9	28	12	1	10	17	103	75	10	yes
55	26	30	10	7	6	0	19	9	0	8	26	450	0	0	yes
67	30.8	30	8	17.5	5	5	21	14	0	8	48	5	200	28	yes
34	24.4	30	8	6	2	1	19	9	1	7	26	40	350	36	yes
46	25.7	15	9	7.5	6	2	14	13	0	15	15	50	0	3	yes
18	26	30	6	7	7	0	7	4	0	7	45	75	210	27	no
35	18.4	30	4	14	11	0	0	5	0	12	26	35	75	19	no
50	22.1	27	5	35	9	4	16	9	0	12	43	85	15	11	**N/A**
59	30.7	30	6	12.5	5	3	7	6	0	14	11	238	350	31	yes
45	21.6	27	6	15	9	4	19	9	0	12	26	80	75	18	**N/A**
41	25.7	30	6.5	17.5	6	0	3	5	0	11	32	219	200	25	no
40	24.1	30	7	12.5	22	7	17	10	2	15	16	356	0	9	yes
61	28	16	6	20	1	0	0	3	0	3	43	8	75	13	yes
58	24.4	15	7	2.5	3	3	6	5	0	4	34	30	200	25	no
26	23.4	27	6	20	1	7	28	3	0	10	46	29	200	25	no
22	21.3	30	4	11	6	2	0	7	0	14	14	186	350	25	no
41	28.3	30	8	17.5	6	1	7	4	0	16	30	183	120	23	yes
27	22.2	27	7	1	15	15	19	16	0	19	19	35	45	9	yes
55	25.7	30	8	7	4	0	24	12	0	6	39	32	210	24	yes
31	39.1	27	8	20	11	1	0	10	4	17	28	15	0	18	no

Supplementary Table 3. Agglomeration Schedule results involving all 100 cases with medians replacing missing data.

Clustering Stages	Agglomeration Coefficients
1	4.5
2	27.405
3	73.15
4	199.198
5	351.698
6	507.568
7	759.898
8	1019.843
9	1285.843
10	1561.163
11	1859.468
12	2177.048
13	2497.173
14	2849.383
15	3203.103
16	3558.213
17	3913.708
18	4286.583
19	4673.053
20	5068.053
21	5473.398
22	5913.358
23	6377.063
24	6846.688
25	7338.293
26	7834.793
27	8334.813
28	8837.193
29	9384.023
30	9935.503
31	10549.673
32	11164.818
33	11794.663
34	12449.343

35	13104.651
36	13763.844
37	14449.559
38	15186.559
39	15944.872
40	16705.092
41	17468.46
42	18233.295
43	19051.212
44	19869.327
45	20735.827
46	21654.13
47	22616.41
48	23665.035
49	24722.83
50	25788.75
51	26868.208
52	27978.655
53	29105.405
54	30238.342
55	31382.697
56	32607.619
57	33841.427
58	35216.632
59	36662.257
60	38122.212
61	39680.768
62	41250.934
63	42856.768
64	44497.409
65	46187.731
66	47882.823
67	49656.458
68	51440.194
69	53480.401
70	55594.706
71	57870.436
72	60190.87
73	62662.825

74	65425.756
75	68830.452
76	72328.02
77	76202.441
78	80132.768
79	84081.219
80	88309.407
81	93027.875
82	98166.728
83	104251.456
84	110694.978
85	120303.355
86	131673.689
87	143103.984
88	157659.51
89	173324.516
90	189182.069
91	212989.282
92	247488.062
93	296457.169
94	351935.706
95	464255.858
96	630913.946
97	832199.725
98	1287984.661
99	2207046.923

Supplementary Table 4. Agglomeration Schedule results involving the 90 cases without missing data.

Clustering Stages	Agglomeration Coefficients
1	4.5
2	117.12
3	272.99
4	516.695
5	769.025
6	1028.97
7	1294.97
8	1570.29
9	1868.595
10	2186.175
11	2538.385
12	2892.105
13	3247.215
14	3633.685
15	4039.03
16	4478.99
17	4942.695
18	5412.32
19	5903.925
20	6400.425
21	6900.445
22	7402.825
23	7949.655
24	8501.135
25	9119.343

26	9749.188
27	10403.87
28	11059.18
29	11728.68
30	12414.4
31	13174.62
32	13937.99
33	14702.82
34	15516.86
35	16334.78
36	17152.89
37	18019.39
38	18937.7
39	19899.98
40	20921.92
41	21970.54
42	23028.34
43	24094.26
44	25169.32
45	26248.78
46	27359.22
47	28485.97
48	29618.91
49	30763.27
50	32138.47
51	33548.05
52	34978
53	36423.63
54	38008
55	39623.18

56	41313.51
57	43008.6
58	44724.9
59	46498.54
60	48612.84
61	50888.57
62	53203.66
63	55675.62
64	58436.12
65	61199.05
66	64165.68
67	67559.81
68	70964.5
69	74838.92
70	78956.5
71	83674.96
72	88813.82
73	94386.41
74	100829.9
75	109433.5
76	119475.5
77	130902.6
78	143734.1
79	158289.6
80	174258
81	194852.1
82	224043.6
83	269508.9
84	324987.5
85	435442.7

86	590894.6
87	783924.9
88	1182905
89	2070050
